# Awareness of obstetric danger signs among pregnant women in the Democratic Republic of Congo: evidence from a nationwide cross-sectional study

**DOI:** 10.1186/s12905-021-01234-3

**Published:** 2021-02-26

**Authors:** Dalau Mukadi Nkamba, Gilbert Wembodinga, Pierre Bernard, John Ditekemena, Annie Robert

**Affiliations:** 1 Kinshasa School of Public Health, Faculty of Medicine, University of Kinshasa, Kinshasa, Democratic Republic of Congo; 2 Pôle D’Épidémiologie et Biostatistique, Institut de Recherche Expérimentale et Clinique (IREC), Université Catholique de Louvain (UCLouvain), Clos Chapelle-aux-champs, 30 bte B1.30.13, 1200 Brussels, Belgium; 3 Pôle de Gynécologie et Obstétrique, Institut de Recherche Expérimentale et Clinique (IREC), Université Catholique de Louvain (UCLouvain), Brussels, Belgium

**Keywords:** Awareness, Obstetric danger signs, Pregnancy, DRC

## Abstract

**Background:**

Poor awareness of obstetric danger signs is a major contributing factor to delays in seeking obstetric care and hence to high maternal mortality and morbidity worldwide. We conducted the current study to assess the level of agreement on receipt of counseling on obstetric danger signs between direct observations of antenatal care (ANC) consultation and women’s recall in the exit interview. We also identified factors associated with pregnant women’s awareness of obstetric danger signs during pregnancy in the Democratic Republic of Congo (DRC)

**Methods:**

We used data from the 2017–2018 DRC Service Provision Assessment survey. Agreement between the observation and woman’s recall was measured using Cohen’s kappa statistic and percent agreement. Multivariable Zero-Inflated Poisson (ZIP) regression was used to identify factors associated with the number of danger signs during pregnancy the woman knew.

**Results:**

On average, women were aware of 1.5 ± 1.34 danger signs in pregnancy (range: 0 to 8). Agreement between observation and woman’s recall was 70.7%, with a positive agreement of 16.9% at the country level but ranging from 2.1% in Bandundu to 39.7% in Sud Kivu. Using multivariable ZIP analysis, the number of obstetric danger signs the women mentioned was significantly higher in multigravida women (Adj.IRR = 1.38; 95% CI: 1.23–1.55), in women attending a private facility (Adj.IRR = 1.15; 95% CI: 1.01–1.31), in women attending a subsequent ANC visit (Adj.IRR = 1.11; 95% CI: 1.01–1.21), and in women counseled on danger signs during the ANC visit (Adj.IRR = 1.19; 95% CI: 1.05–1.35). There was a regional variation in the awareness of danger signs, with the least mentioned signs in the middle and the most in the eastern provinces.

**Conclusions:**

Our findings indicated poor agreement between directly observed counseling and women’s reports that counseling on obstetric danger signs occurred during the current ANC visit. We found that province of residence**,** provision of counseling on obstetric danger signs, facility ownership, gravidity and the number of ANC visits were predictors of the awareness of obstetric danger signs among pregnant women. These factors should be considered when developing strategies aim at improving women’s awareness about obstetric danger signs in the DRC

## Background

Pregnancy and childbirth related complications are leading causes of maternal mortality worldwide [1]. From World Health Organization (WHO) estimations, about 295,000 women died in 2017 as a result of complications during and following pregnancy and childbirth [2]. Most of these deaths (66%) occurred in Sub-Saharan Africa because of delay in seeking care, delay in reaching a healthcare facility, and delay in receiving appropriate care once at the facility. Poor knowledge about obstetric danger signs is a major contributing factor to delay in seeking obstetric care, and hence to high maternal morbidity and mortality in low and middle income countries [3, 4]. Major obstetric danger signs during pregnancy include severe vaginal bleeding, severe headache, preterm labor, rupture of membrane before onset of labor, epigastric pain, severe abdominal pain, convulsions, blurred vision, and fever [5]. Studies in sub-Saharan African countries indicate poor awareness of these signs among pregnant women and communities [6–8].

According to WHO, the maternal mortality in the Democratic Republic of Congo (DRC) dropped from 760 deaths per 100,000 live births in 2000 to 473 in 2017, a decrease of 38% [2]. The country failed to reach the Millennium Development Goal target despite an increase in institutional deliveries and skilled attendance from 70 and 74% in 2007 to 81.5% and 85.2% in 2018, respectively [9, 10].

In 2018, delay in seeking obstetric care accounted for 34% of maternal deaths, according to the DRC’s maternal death surveillance system [11]. Maternal mortality could be reduced by empowering women with knowledge on the obstetric danger signs and promoting timely seeking of emergency obstetric care (EmOC). The focused antenatal care (FANC) guidelines in the DRC recommend that antenatal care (ANC) providers counsel all pregnant women and their families about obstetric danger signs at each visit, as obstetric complications can be unpredictable [12].

A services provision assessment survey was conducted in the DRC (DRC-SPA) from 2017 to 2018 to evaluate the quality of healthcare services nationwide [13]. The DRC-SPA survey collected data through direct observations of ANC consultations, and exit interviews with women receiving care at the facility. The direct observations assessed whether the provider performed any ANC counseling, including counseling on obstetric danger signs. During the exit interview, women whose consultation was observed were asked to recall ANC interventions they received during the visit.

Examining the level of agreement between the direct observation of the ANC consultation and the woman’s recall of the consultation in the exit interview is of utmost importance in understanding the quality of ANC [14]. Studies in Haiti, Malawi, and Senegal showed little agreement between the direct observation of counseling during ANC visit and a woman’s recall [14]. In the DRC however, little is known regarding such agreement with respect to the counseling on obstetric danger signs during pregnancy. Although the DRC-SPA report indicated poor knowledge of obstetric danger signs among women receiving ANC, the report failed to identify reasons of such poor knowledge [13]. To improve maternal health seeking behavior, identifying factors that affect knowledge of obstetric danger signs is of paramount priority [6, 14].

Therefore, we aimed (1) to assess the level of agreement on receipt of ANC counseling on obstetric danger signs between direct observations of ANC consultation and women’s recall in the exit interview; and (2) to identify factors associated with the knowledge of obstetric danger signs among pregnant women nationwide.

## Methods

### Data source

We used data from the 2017–2018 DRC-SPA survey, a nationwide facility-based and cross-sectional study. The survey was implemented by the Kinshasa School of Public Health (KSPH) with a technical assistance from Inner City Fund International through funding from the United States Agency for International Development (USAID).

### Sample size and sampling procedure

**Fig. 1:**
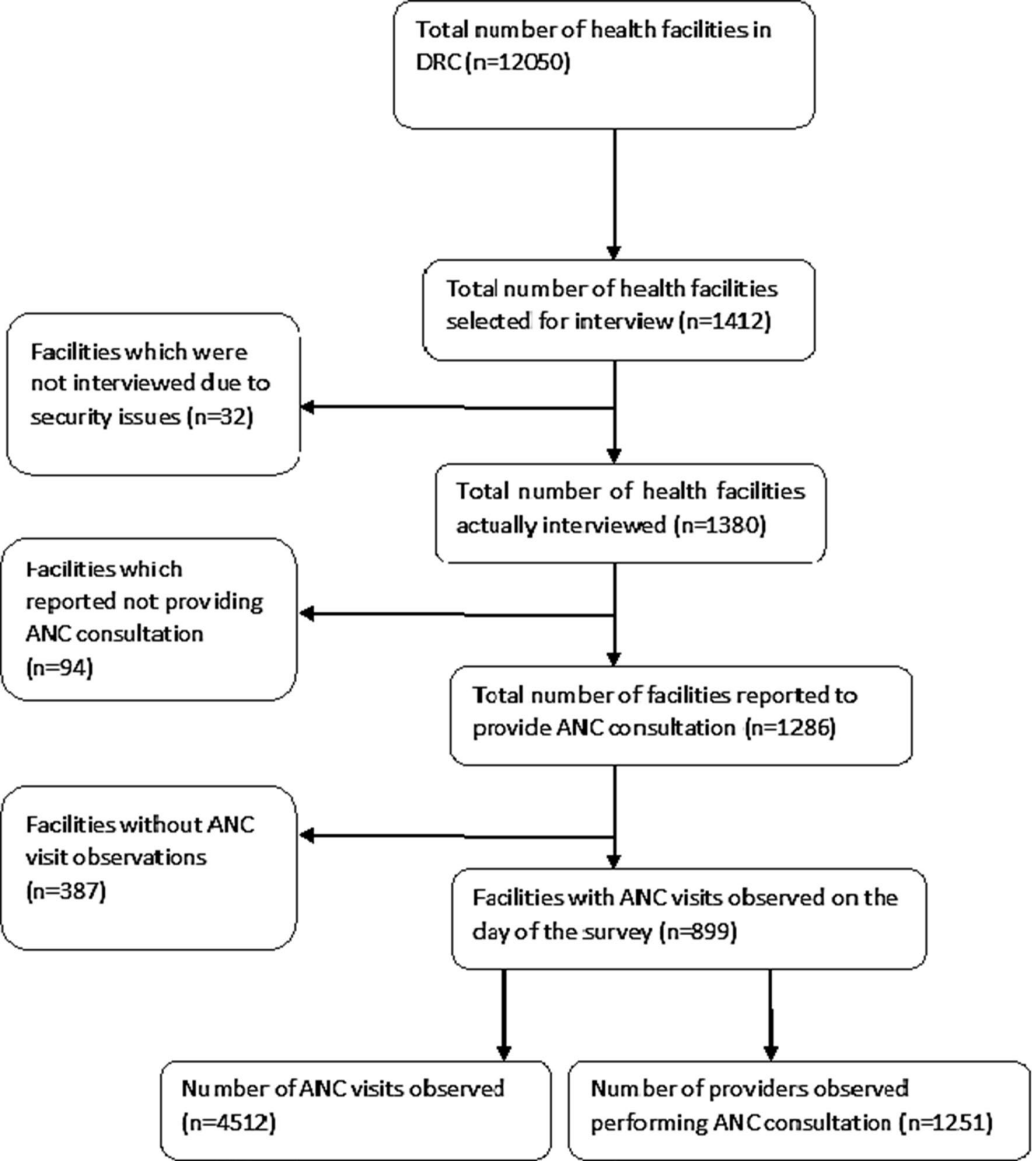
Selection procedure for the sampling units included in this analysis

The sampling process for the DRC-SPA survey has been described in detail elsewhere [13]. Briefly, the survey was conducted in a stratified sample of 1412 health facilities. From these health facilities, 32 could not be reached due to their location in areas where there were still armed conflicts. As a result, 1380 health facilities were surveyed, of which only 899 received an antenatal visit during the study period. Our analysis is based on these 899 health facilities including 5 tertiary hospitals, 556 secondary and 338 primary health centres (PHCs). In each health facility, five women per ANC provider were included in the survey with a maximum of 15 women per facility. The final sample size was 4512 women (Fig. 1). The data were collected between October 16, 2017 and November 24, 2017 in Kinshasa, and from January 18 to April 20, 2018 in the rest of the DRC.

Four data collection tools were used in this survey: (1) facility inventory questionnaire; (2) healthcare provider interview questionnaire; (3) checklist for direct observation of services for sick children, family planning, and ANC; and (4) exit interview questionnaires for caretakers of sick children, for women receiving ANC, and for women receiving family planning whose consultations were observed.

For each pregnant woman, a data collector observed the provider conducting the ANC consultation using the ANC direct observation checklist. The data collector observed whether the provider asked about, counseled, or discussed any of seven main danger signs of pregnancy complications including vaginal bleeding, headache or blurred vision, swollen face or hands, reduced or no fetal movement, tiredness or breathlessness, cough or difficulty breathing, and fever.

The exit interview was conducted immediately after the ANC consultation. During the exit interview, data collectors asked women about their visit, including the counseling they received, whether they received the counseling on danger signs in the current visit, in the current and a previous visit, in a previous visit only, or not at all. We considered a woman’s response that counseling was performed in the current visit as indicating that counseling on any of the danger signs was performed during the ANC consultation. Women were also asked to list the danger signs of pregnancy complications that they know. The eight possible signs that the woman could mention are convulsions and the seven danger signs used in the direct observation checklist.

### Operational definitions

We used the following definitions:

Positive agreement: both the direct observation of ANC consultation and woman’s report agreed that the counseling was performed.

Negative agreement: both the direct observation of ANC consultation and woman’s report agreed that the counseling was not performed.

### Data processing and management

For our analysis, we merged four datasets (facility inventory, provider interview, direct observation of ANC consultation, and exit interview datasets) into one dataset using the provider and facility identifiers of each dataset. The unit of analysis of the merged dataset was an individual woman.

Secondary and tertiary health facilities were grouped into one category named “hospitals”, as they are all used as referral units by PHCs. We combined the woman’s knowledge about a given sign, and the direct observation of the counseling to get a variable with four categories: (1) woman counseled about the sign and mentioned the sign; (2) woman counseled about the sign but did not mention the sign; (3) woman not counseled about the sign but mentioned the sign; (4) woman not counseled about the sign and did not mention the sign. Provinces were grouped into middle, including Kasai Occidental and Kasai Oriental; northern provinces including Equateur and Oriental; western including Bas Congo, Bandundu and Kinshasa; southern including Katanga; and eastern including Maniema, Nord Kivu and Sud Kivu.

### Data analysis

Data were analyzed using Stata 14.1 software (StataCorp, College Texas). All analyses were weighted using sampling weights provided in the DRC-SPA datasets. We used the “svy” command to account for the sampling design. Continuous variables were summarized using weighted mean and standard deviation (± SD) if normally distributed, and median and interquartile range (IQR) for non-normal distributions. Categorical variables were summarized using weighted proportions. The percent agreement and the Cohen’s kappa (κ) statistic were used to measure the level of agreement between the woman’s recall from the exit interview and the direct observation of the counseling during the ANC visit. A Cohen’s kappa of zero or less was considered as no agreement, 0.01 to 0.20 as a slight agreement, 0.21 to 0.40 as a fair agreement, 0.41 to 0.60 as a moderate agreement, 0.61 to 0.80 as a substantial agreement, and 0.81 to 1.0 as a perfect agreement [15].

We computed the proportion of positive agreement by dividing the number of pregnant women who agreed with the direct observation that the counseling was performed during the ANC visit, by the total number of pregnant women. The proportion of negative agreement was obtained by dividing the number of pregnant women who agreed with the observation that the counseling was not performed during the ANC visit, by the total number of pregnant women. The overall agreement was computed as the sum of both positive and negative agreements.

Our dependent variable was the number of danger signs the pregnant woman mentioned from the possible eight signs. The dependent variable was measured as a count score of these signs ranging from 0, for women who did not mention any sign, to 8 for those who mentioned all the 8 signs.

We explored several regression models including Poisson regression model, zero-inflated Poisson (ZIP) regression model, negative binomial regression model, and zero-inflated binomial regression model. Based on a goodness of fit test we used ZIP regression model.

Independent variables included women’s characteristics (scholarship, gestational age, number of previous ANC visits for the current pregnancy, whether the pregnancy was the first, and whether the woman received counseling on danger sings); provider characteristics (qualification, receipt of in-service training in ANC counseling in the past 24 months); and facility characteristics (location, facility type, facility ownership, province, mean number of ANC visits per day).

Quantitative independent variables such as the mean number of ANC visits per health facility per day were standardized to avoid scale dependence. All independent variables with a P-value less than 0.25 in univariable regression were candidates for multivariable analysis. If a strong correlation was noticed between two independent variables, one amongst the two was eliminated to avoid multicollinearity. We checked multicollinearity among independent variables by estimating the variance inflation factor [16]. All variance inflation factor values were less than 10, so there was no multicollinearity.

Results are presented as crude or adjusted incidence risk ratios (Adj.IRR) with 95% confidence intervals (95% CI). The statistical significance level was set to 0.05.

## Results

Within each province, more than half of the pregnant women were in rural settings, except in Kinshasa where 88.6% were in an urban area. The median (IQR) gestational age at inclusion ranged from 22 (18–28) weeks in Kasaï Occidental to 30 (25–36) weeks in Bas Congo (Table 1). Counseling on any danger signs was observed in 35.0% of ANC visits, and 28.4% of women reported receiving the counseling during the current visit.

**Table 1:** Characteristics of interviewed pregnant women attending antenatal care in 2017–2018 in the DRC, within each of the 11 provinces

Characteristic	Provinces										
	Bandundu (n = 335)	Bas congo (n = 119)	Equateur (n = 696)	Kasai Occidental (n = 183)	Kasai Oriental (n = 257)	Katanga (n = 727)	Kinshasa (n = 352)	Maniema (n = 92)	Nord Kivu (n = 446)	Sud Kivu (n = 885)	Oriental (n = 420)
Scholarship—%											
Primary or less	47.0	40.7	65.2	56.4	55.8	59.9	24.4	37.1	61.4	54.8	77.2
Secondary or technical	53.0	59.3	34.8	43.6	44.2	40.1	75.6	62.9	38.6	45.2	22.8
Location—%											
Rural	98.2	86.8	96.5	77.2	59.4	66.7	11.4	58.4	75.9	82.7	69.6
Urban	1.8	13.2	3.5	22.8	40.6	33.3	88.6	41.6	24.1	17.3	30.5
Gestational age—weeks											
Median	24	30	26	22	24	28	26	27	28	28	28
IQR	20–28	25–36	20–31	18–28	16–30	24–32	20–30	20–30	21–32	24–32	22–32
Mean (± SD)	25 (± 7)	29 (± 7)	26 (± 7)	23 (± 7)	23 (± 9)	27 (± 7)	25 (± 7)	26 (± 7)	27 (± 8)	29 (± 6)	26 (± 6)
< 16 weeks —%	7.7	3.1	6.2	10.3	22.5	5.9	8.1	5.2	5.5	0.9	4.4
First pregnancy—%	25.8	29.7	16.9	31.3	18.1	25.7	34.3	21.4	25.9	37.3	18.7
First ANC visit—%	43.8	40.2	62.7	63.3	61.4	42.5	42.6	44.5	50.9	57.9	54.5

*IQR* interquartile range

There was a fair agreement between the observed counseling on danger signs and the women’s report from the exit interview. The percent agreement was 70.7% (95% CI: 67.5–73.7%), with a positive agreement of 16.9% (95% CI: 13.0–21.6%). Agreement varied among provinces, ranging from = κ-0.107 in Kasaï Occidental to = κ0.387 in Bas Congo. There were also disparities in the percent of positive agreement among provinces, ranging from 2.1% (1.0–4.5%) in Bandundu to 39.7% (23.9–57.9%) in Sud Kivu (Table 2).

**Table 2:** Percentage of observed counseling on obstetric danger signs, and percentage of women reporting receiving the counseling, together with reported percent agreement and Cohen’s κ statistics in each province of the DRC

Province of residence	Providers observed counseling on danger signs % (95% CI)	Women reported receiving the counseling %(95% CI)	Percent agreement on counseling^a^	Percent of positive agreement on counseling^b^	Cohen's κ
Bandundu	9.5 (5.9–14.7)	14.9 (9.4–22.8)	79.9 (71.2–86.5)	2.1 (1.0–4.5)	0.052
Bas Congo	34.2 (14.2–62.1)	38.9 (18.8–63.6)	75.0 (62.1–84.6)	24.1 (6.7–58.3)	0.387
Equateur	22.1 (16.2–29.3)	26.6 (21.6–32.4)	68.7 (61.7–74.9)	8.7 (5.7–13.0)	0.174
Kasai Occidental	30.9 (19.6–45.2)	14.5 (8.2–24.3)	62.2 (50.6–72.6)	3.9 (1.5–9.7)	− 0.107
Kasai Oriental	16.0 (9.9–24.7)	10.8 (6.1–18.4)	78.5 (69.3–85.5)	2.6 (1.2–5.8)	0.106
Katanga	31.1 (22.9–40.6)	25.6 (17.2–36.3)	69.2 (60.9–76.5)	12.9 (7.3–22.0)	0.235
Kinshasa	14.9 (8.9–24.0)	24.1 (16.1–34.5)	70.7 (61.1–78.7)	4.8 (2.6–8.7)	0.069
Maniema	42.8 (23.4–64.6)	35.3 (25.0–47.2)	65.4 (50.4–77.9)	21.8 (10.2–40.4)	0.326
Nord Kivu	69.8 (55.7–80.9)	37.9 (24.5–53.5)	57.8 (44.6–69.9)	32.7 (19.8–48.9)	0.238
Sud Kivu	59.3 (43.7–73.2)	44.2 (27.4–62.4)	75.8 (65.1–84.0)	39.7 (23.9–57.9)	0.383
Oriental	21.0 (13.8–30.7)	19.2 (13.5–26.6)	70.9 (62.3–78.1)	5.5 (2.9–10.2)	0.168
Overall	34.7 (30.7–38.9)	28.4 (24.1–33.1)	70.7 (67.5–73.7)	16.9 (13.0–21.6)	0.219

^a^Agreement between the observation of counseling on danger signs and the woman’s report in the exit interview

^b^Both the observation of ANC consultation and woman’s report agreed that the counseling was performed

**Fig. 2:**
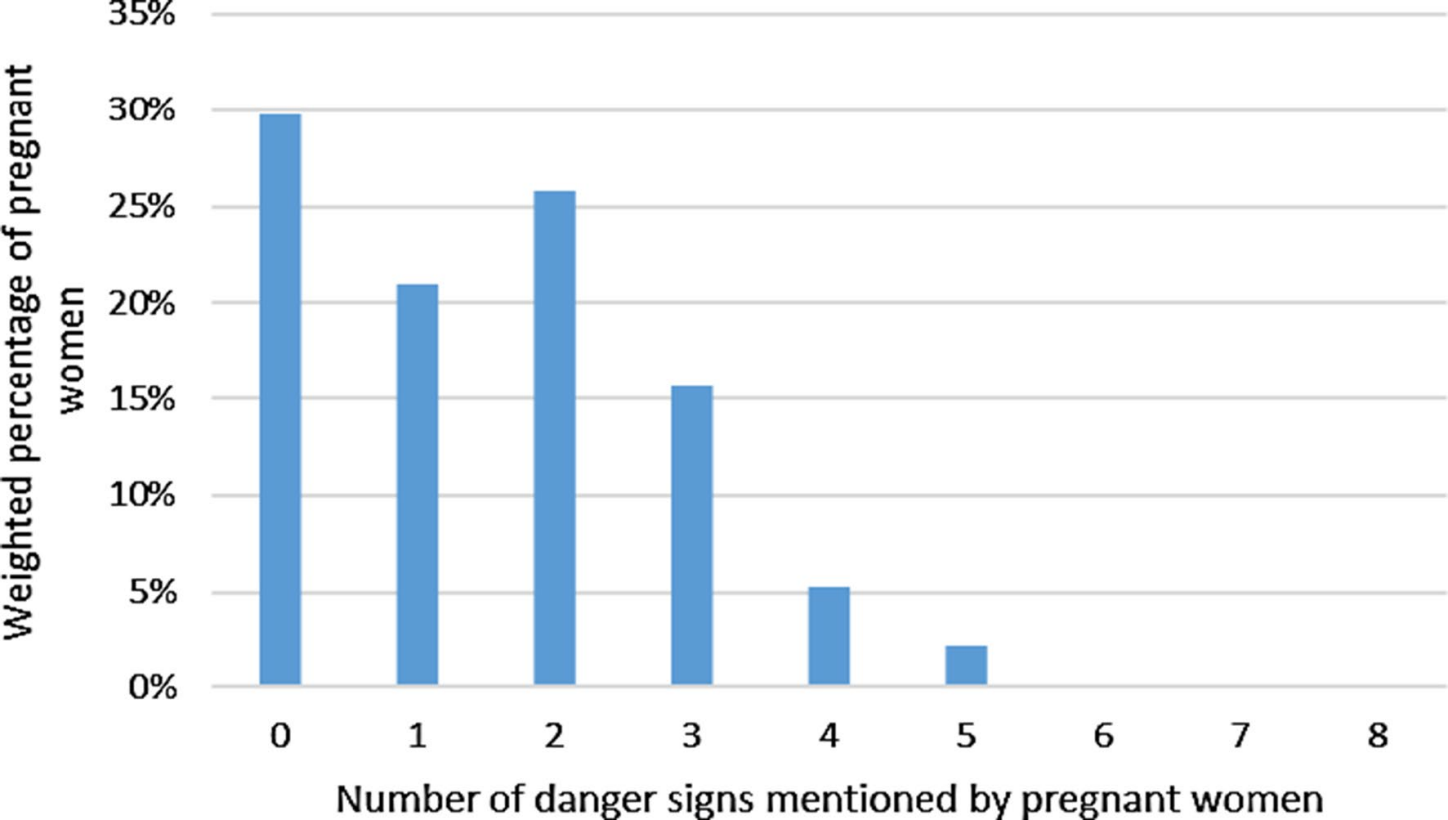
The number of dangers signs mentioned by pregnant women during the exit interview after antenatal care visit in the DRC. Mean (± SD) number of signs mentioned by pregnant women was 1.53 (± 1.34), with a median (IQR) of 1 (0–2)

More than two thirds of pregnant women mentioned at least one danger sign, half mentioned at least two signs, and almost no woman reported all of the eight signs. The median (IQR) number of signs reported was 1 (0–2) (Fig. 2).

**Fig. 3:**
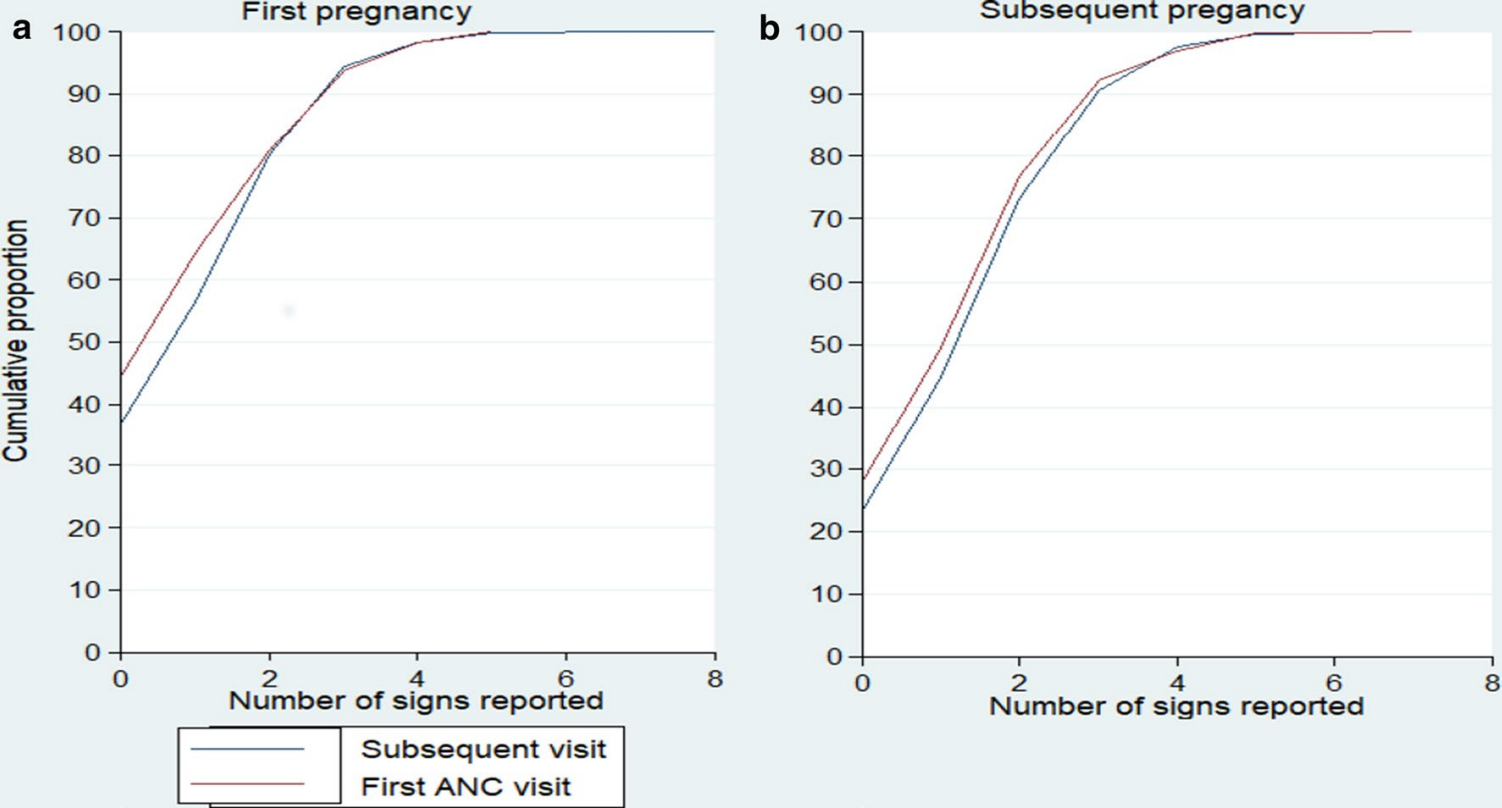
Cumulative distribution of the number of danger signs reported by pregnant women during the exit interview after ANC visit, by gravidity in the DRC. **a** Related to first pregnant women, n = 1195 of which 664 were in their first ANC visit and 531 were in a subsequent visit. **b** Related to subsequent pregnant women, n = 3317 of which 1708 were in their first ANC visit and 1609 were in a subsequent visit

Among primigravida women, 44.4% (95% CI: 36.7–52.4%) of those attending a first ANC visit and 36.9% (95% CI: 29.8–44.7%) of those attending a subsequent ANC visit could not mention any danger sign. Among multigravida women, 28.0% (95% CI: 24.6–31.7%) of those attending a first ANC visit and 23.3% (95% CI: 19.9–27.0%) of those attending a subsequent ANC visit could not mention any danger sign (Fig. 3).

**Fig. 4:**
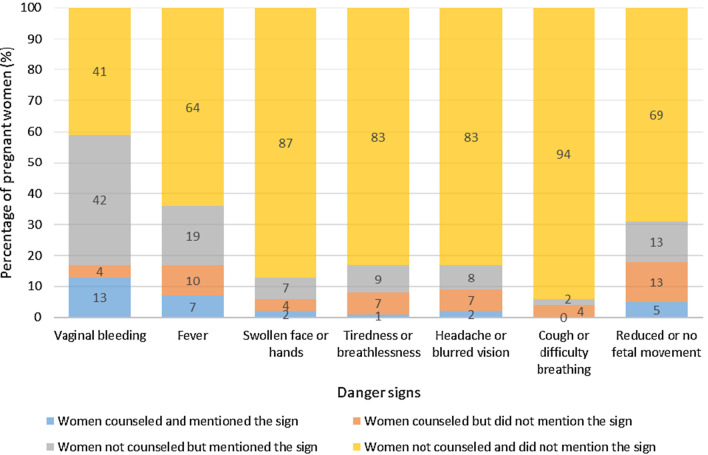
Percentage of women counseled on obstetric danger signs, and percentage of women reporting each sign in the DRC

Vaginal bleeding was the most mentioned sign (55%) and reduced- or no fetal movement was the sign counseled on the most (18%). Although 9% of women received counseling on headache and blurred vision, only 2% mentioned these sign (Fig. 4).

After multivariable ZIP analysis, the number of obstetric danger signs the women mentioned was significantly higher in multigravida women (Adj.IRR = 1.38; 95% CI: 1.23–1.55), in women attending a private facility (Adj.IRR = 1.15; 95% CI: 1.01–1.31), in those attending a subsequent ANC visit (Adj.IRR = 1.11; 95% CI: 1.01–1.21), in women counseled on danger signs during the ANC visit (Adj.IRR = 1.19; 95% CI: 1.05–1.35), and in women attending ANC visit in eastern (Adj.IRR = 2.04; 95% CI: 1.64–2.55), southern (Adj.IRR = 1.69; 95% CI: 1.32–2.17), or northern (Adj.IRR = 1.49; 95% CI: 1.21–1.83) provinces as compared to provinces in the middle part of the country (Table 3).

**Table 3:** Factors associated with the knowledge of obstetric danger signs among pregnant women attending antenatal care in the DRC, using weighted Zero inflated Poisson regression model

Factors	Mean (± SD)	Crude IRR (95% CI)	*p* value	Adjusted IRR (95% CI)	*p* value
Location			0.85		
Urban	1.50 ± 1.43	1.02 (0.80–1.31)			
Rural	1.55 ± 1.31	1		1	
First ANC visit			0.09		0.03
Yes	1.46 ± 1.35	1		1	
No	1.61 ± 1.33	1.09 (0.99–1.21)		1.11 (1.01–1.21)	
Facility type			0.33		
PHCs	1.54 ± 1.37	1			
Hospital	1.51 ± 1.24	0.94 (0.83–1.06)			
Gestational age			0.11		0.24
< 20 weeks	1.36 ± 1.29	1		1	
≥ 20 weeks	1.57 ± 1.35	1.14 (0.97–1.32)		0.92 (0.81–1.06)	
First pregnancy			0.004		< 0.001
Yes	1.25 ± 1.32	1		1	
No	1.63 ± 1.33	1.23 (1.07–1.42)		1.38 (1.23–1.55)	
Scholarship			0.58		
Primary or less	1.50 ± 1.35	1			
Secondary and above	1.58 ± 1.32	1.03 (0.93–1.14)			
Type of health providers			0.51		
Midwife	1.54 ± 1.34	1			
Physician	1.37 ± 1.34	0.93 (0.75–1.15)			
Clinic ownership			0.012		0.05
Private	1.73 ± 1.40	1.24 (1.05–1.47)		1.15 (1.01–1.31)	
Public	1.41 ± 1.28	1		1	
Mean number of ANC visits per day	1.53 ± 1.34	1.06(1.01–1.11)	0.02	1.04 (0.98–1.09)	0.20
Provider counseled on danger signs during ANC visit			< 0.001		0.008
No	1.32 ± 1.25	1		1	
Yes	1.93 ± 1.41	1.38 (1.18–1.61)		1.19 (1.05–1.35)	
Women attended by a provider who received in-service training in ANC counseling within the previous 2 years			0.65		
No	1.53 ± 1.38	1			
Yes	1.54 ± 1.28	0.97 (0.84–1.11)			
Provinces of residence			0.00		< 0.001
Middle	0.90 ± 1.08	1		1	
Eastern	2.15 ± 1.30	2.09 (1.68–2.59)		2.04 (1.64–2.55)	
Southern	1.40 ± 1.45	1.66 (1.29–2.13)		1.69 (1.32–2.17)	
Western	1.13 ± 1.18	1.18 (0.93–1.49)		1.22 (0.97–1.53)	
Northern	1.38 ± 1.22	1.42 (1.18–1.73)		1.49 (1.21–1.83)	

*IRR* Incidence risk ratio, *PHCs* Primary Health Centres, *ANC* antenatal care

*Whether ANC provider encouraged women to ask questions during the ANC visit

## Discussion

Poor knowledge about obstetric danger signs can adversely affect women’s preparedness for pregnancy complications. We conducted the current study to assess the level of agreement between the direct observation of ANC counseling on obstetric danger signs and the women’s recall; and to identify factors associated with the knowledge of obstetric danger signs during pregnancy nationwide in the DRC.

Our study revealed that women were aware of 1.5 danger signs on average, while the mean number of signs health providers counseled on was 1 out of seven possible. Only 35% of directly observed ANC consultations provided counseling on any obstetric danger sign. These observations suggest that poor knowledge about obstetric danger signs may result from inadequate counseling.

The prevalence of counseling on obstetric danger signs in the DRC is lower than reported from Malawi in 2018 (54%) [14]. The failure to adequately counsel women on danger signs in the DRC may be a consequence of providers’ knowledge gaps, lack of supervision, or motivation. However, the etiology of this failure needs further investigation. Although heavy ANC workload could be another explanation for the observed poor counselling on obstetric danger signs, this is unlikely given that health facilities included in this study had relatively low volume of ANC attendees on the day of the survey, as half of them received less than 10 ANC visits.

The proportion of pregnant women recalling receiving counseling in the DRC was lower than the proportion reported in Malawi (51.3%) [14]. This may be explained by the higher prevalence of counseling on danger signs in Malawi as compared to the DRC. Consistent with studies from other sub-Saharan African countries [6, 7, 14], we found that vaginal bleeding was the most known danger sign. However, knowledge of this danger sign may not be a direct result of ANC counseling since a higher percentage of women mentioned the sign without counseling than women during the directly observed ANC consultation. This finding suggests that women in the DRC may have become aware of danger signs from a source other than their ANC providers. This was supported by the fact that 35% of primigravida women in first ANC visit were not counseled about vaginal bleeding but mentioned the sign during their exit interview.

It is worrisome that signs like headache or blurred vision and swollen face or hands, which are indicative of hypertensive disorders in pregnancy, were mentioned only by 10% of pregnant women, even though this obstetric complication is one of the most common causes of maternal deaths in the DRC. This gap in understanding may result from the common, non-specific nature of some of these signs. As reported in a study from Tanzania [7], pregnant women may consider signs such as headache and tiredness as normal events during pregnancy, and therefore do not consider these as danger signs. Therefore, ANC providers should enhance counseling on these obstetric danger signs that pregnant women inadvertently regard as normal events.

Every pregnant woman is at risk of developing pregnancy-related complications, some of which are unpredictable [5]. WHO ANC guidelines recommend that counseling on danger signs of pregnancy should be performed during each ANC visit [17], to allow appropriate EmOC seeking actions once the signs are recognized. Our study indicates that although more than 90% of pregnant women in the DRC attend at least one ANC visit [10], they do not get enough information on obstetric danger signs and may not benefit optimally from ANC services. A study from Tanzania reported an association between women’s knowledge of obstetric danger signs during pregnancy and their subsequent EmOC seeking behavior [7].

Our study implies a missed opportunity to empower women on safe pregnancy and help them make prompt decision once obstetric complications arise either before. The low awareness of obstetric danger signs in the DRC coupled with poor counseling may contribute to the delay in seeking appropriate EmOC, which in turn is likely to increase the risk of maternal mortality. Therefore, it is not unexpected that 34% of maternal deaths in the DRC result from a delay in seeking appropriate EmOC for an obstetric emergency [11].

Although the exit interview with pregnant women occurred immediately following the ANC consultation, we found a low level of agreement between the observation of counseling and the women’s recall that the counseling was performed, with disparities between provinces. Provinces such as Nord Kivu and Sud Kivu showed the highest level of counseling and the highest level of positive agreement, as compared to other provinces. Overall, one-fifth of women reported not receiving counseling while the ANC consultation was observed providing the counseling. This may be due to the insufficient emphasis on informing women about danger signs during ANC visits, or because the women did not pay enough attention to the counseling, or because the counseling was given in a group instead of individually.

Although counseling is known to improve understanding of danger signs, we found poor awareness of these signs and poor positive agreement in the DRC. The study highlights the need to improve both the quantity of counseling (every woman should be counseled) but also to improve the quality of counseling. We are uncertain of the best approach to improve awareness of danger signs. However, we speculate that individual counseling of mothers about danger signs, in contrast to attempts to educate groups of mothers, may improve awareness. Although this approach may not be appropriate in all settings, it would be possible in this area of the DRC because of a low volume of ANC attendees. In addition, other approaches such as use of visual aids, repetitive messages outside of the clinic through community health workers or mobile health (mHealth), and knowledge reviews at the end of clinic visit [18–20] should be tested in subsequent improvement efforts.

When investigating the factors associated with knowledge of obstetric danger signs among women, several important factors were identified. The level of knowledge about obstetric danger signs was found to be higher among multigravida than in primigravida women. This finding aligns with other studies from sub-Saharan countries [8, 21]. This could be attributed to the fact that multigravida women have learned from their own experiences, especially if they previously experienced obstetric complications.

Having more ANC visits was also found to significantly increase the number of obstetric danger signs the women mentioned. Similar findings were reported in studies from Malawi, Senegal, and Tanzania [7, 14, 22]. ANC is an opportunity to provide health information including counseling on obstetric danger signs. The more ANC visits for a woman, the more exposition to health information, and so the higher the knowledge about obstetric danger signs.

Women attending ANC in private health facilities were significantly more knowledgeable compared to their counterpart in public health facilities. Our findings corroborate some previous studies in the DRC highlighting a better quality of emergency obstetric care in private than in public facilities [23].

Knowledge of obstetric danger signs during pregnancy was also significantly higher among women in eastern, southern, and northern provinces compared to women in the central region of the country. One possible reason for this finding is that eastern provinces showed a higher prevalence of counseling, with a higher level of positive agreement between the observation of counseling and the women’s report that the counseling occurred. This finding is in line with a study from Senegal and Malawi that reported a significant association between positive agreement and the knowledge of obstetric danger signs [14].

Our study has provided insight into factors that should be included in strategies aimed at improving women’s awareness about obstetric danger signs in the DRC. The information could help design appropriate interventions aimed at decreasing delays in seeking EmOC and related maternal deaths.

The strength of our study was the use of a comprehensive national dataset, suggesting that our findings accurately reflect the situation regarding the quality of counseling on obstetric danger signs in the DRC and, thus, the quality of ANC services. Nonetheless, the study has some limitations. The results of the direct observation of counseling on obstetric danger signs may be inflated due to the Hawthorne effect; health providers might have made an extra effort to give their best quality service at the time when the research team visited the health facility. However, this effect was mitigated by having up to five observations per provider, which may have helped to reduce providers’ awareness of the presence of the data collector.

The DRC-SPA survey did not ask women their source of information about obstetric danger signs and missed information on whether women had previously experienced any obstetric complication. Pregnant women may have learned about the signs elsewhere such as through television or personal experience rather than during the ANC visit. We attempted to mitigate this bias by assessing the knowledge of primigravida women in first ANC visit. Despite the possibility that primigravida pregnant women could have learned about obstetric danger signs elsewhere, the level of knowledge was still quite low. Additional information is needed to further understand the factors associated with a woman’s knowledge of obstetric danger signs. Information such as women’s sources of obstetric danger sign, previous pregnancy complications, or case studies on private practices provision of obstetric danger sign counseling would be helpful next steps to contextualize results found in this analysis.

## Conclusion

Our study showed poor knowledge about obstetric danger signs among pregnant women, and inadequate counseling about these signs during ANC visits in the DRC. The findings also indicated poor agreement between directly observed counseling and women’s reports that counseling on obstetric danger signs occurred during the current ANC visit.

We found that province of residence**,** provision of counseling on obstetric danger signs, facility ownership, gravidity and the number of ANC visits were predictors of the awareness of obstetric danger signs among pregnant women. These factors should be considered when developing strategies aim at improving women’s awareness about obstetric danger signs in the DRC.

## Data Availability

The DRC-SPA data is publically available from the DHS website: http://dhsprogram.com/data/available-datasets.cfm.

## Abbreviations

ANC: Antenatal care
CI: Confidence interval
DHS: Demographic and Health Survey
DRC: Democratic Republic of Congo
EmOC: Emergency obstetric care
HDP: Hypertensive disorder in pregnancy
IQR: Interquartile range
LMIC: Low- and Middle-Income Country
PHCs: Primary health centres
SPA: Service Provision Assessment survey
SD: Standard deviation
